# Physical Frailty Prediction Using Cane Usage Characteristics during Walking

**DOI:** 10.3390/s24216910

**Published:** 2024-10-28

**Authors:** Haruki Toda, Takaaki Chin

**Affiliations:** 1 Robot Rehabilitation Center, The Hyogo Institute of Assistive Technology, Kobe 651-2134, Japan; 2 Hyogo Prefectural Rehabilitation Center, Kobe 651-2134, Japan

**Keywords:** cane, decision tree, frail, frequency analysis, gait, inertial measurement unit, machine learning, older people, root mean square

## Abstract

This study aimed to determine the characteristics of accelerations and angular velocities obtained by an inertial measurement unit (IMU) attached to a cane between older people with and without physical frailty. Community-dwelling older people walked at a comfortable speed using a cane with a built-in IMU. Physical frailty was assessed using exercise-related items extracted from the Kihon Check List. The efficacy of five machine learning models in distinguishing older people with physical frailty was investigated. This study included 48 older people, of which 24 were frail and 24 were not. Compared with the non-frail participants, the older people with physical frailty had a small root mean square value in the vertical and anteroposterior directions and angular velocity in the anteroposterior direction (*p* < 0.001, r = 0.36; *p* < 0.001, r = 0.29; *p* < 0.001, r = 0.30, respectively) and a large mean power frequency value in the vertical direction (*p* = 0.042, r = 0.18). The decision tree model could most effectively classify physical frailty, with an accuracy, F1 score, and area under the curve of 78.6%, 91.8%, and 0.81, respectively. The characteristics of IMU-attached cane usage by older adults with physical frailty can be utilized to effectively evaluate and determine physical frailty in their usual environments.

## 1. Introduction

In Japan, 29.0% of the population is 65 years old or more [1]. Age-related frailty is a factor that causes older adults to require nursing care. Frailty is a condition characterized by a reduction in various physiological reserves, thereby decreasing the capacity to effectively respond to stress [2]. Generally, people who experience frailty show a more rapid functional decline and lower resistance to illness and disability than healthy individuals [3,4]. Early detection of and intervention for frailty are essential for extending older people’s healthy life expectancy.

The Kihon Check List (KCL) is a primary frailty screening tool used in Japan [5]. It is a 25-item, self-administered questionnaire that includes items on exercise, nutrition, oral health, confinement, cognition, and depression. However, questionnaire scoring may have been influenced by bias [6]. In addition, repeated assessments using a questionnaire over a short period may cause fatigue in respondents, thereby affecting the quality and reliability of the responses. Recently, wearable technologies have been used to evaluate the gait performance of older adults [7]. Wearable sensing technologies that can conduct a quantitative assessment daily may be useful for the early detection of frailty among older people. These technologies provide a cost-effective, efficient, and accessible method for standardizing data collection and analysis, both in clinical settings and within the living communities of frail older people [7].

A cane is an application of wearable technology. A cane is one of the most commonly used walking aids for older people. Consequently, previous studies have evaluated movements using an inertial measurement unit (IMU) built into a cane [8,9,10,11]. The IMU can be attached to the cane without any body contact, thus causing no pressure on the device and less strain on the user. In addition, evaluation using cane usage provides a less intrusive method that can continuously monitor changes in gait and balance without requiring daily active participation by the individual. This method reduces response fatigue to frailty assessments and provides a more sustainable approach in long-term monitoring with minimal impact on natural gait patterns. Thus, integrating sensing technology into canes provides a sustainable approach to early frailty detection. Characteristic waveforms observed during cane use [8] enable estimation of walking abilities and terrains using IMU data [12], and the frequency characteristics of acceleration provide further evaluative metrics. Since physical frailty in older adults is generally associated with decreased walking ability [13], changes in cane use may reflect this decline. However, to the best of our knowledge, physical frailty in older adults has not yet been evaluated using an IMU built into a cane.

If the differences in cane usage depending on physical frailty can be clarified and if physical frailty can be accurately classified, daily gait sensing using a cane can quickly detect a decline in the ability to walk due to physical frailty. This study aimed to compare the accuracy of some machine learning techniques in predicting physical frailty among older people based on the characteristics of the IMU sensor built into the cane. By analyzing these characteristics, we determine the most effective machine-learning method for classifying the presence and absence of physical frailty.

## 2. Materials and Methods

### 2.1. Participants

This study recruited older adults living in Harima-cho, Kako-gun, in Hyogo from September to October 2023. The inclusion criteria were as follows: ability to walk independently, a level of cognitive function sufficient to understand the research content, and no severe pain that significantly impacted walking. This study conformed to the principles of the Declaration of Helsinki [14] and obtained approval from the local ethics committee (R2303). All participants signed informed consent forms before data acquisition.

### 2.2. Data Collection

**Figure 1:**
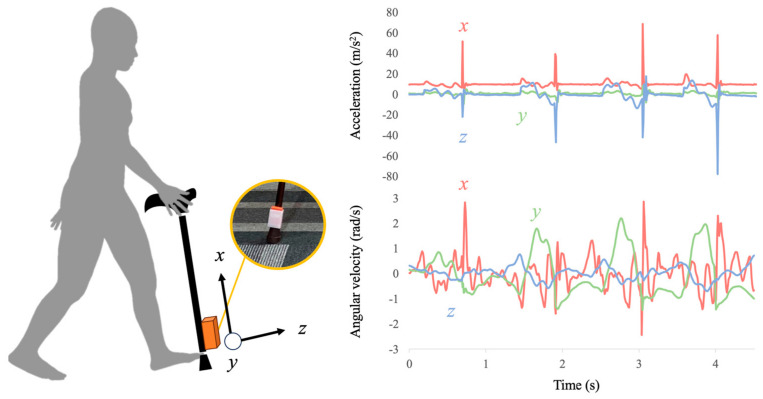
The proposed system using an inertial measurement unit built into a cane to evaluate physical frailty among older adults. The presented data are typical of measurement data.

A cane, which is frequently used by older adults daily, was used for the measurements. An IMU (MTw; Xsens Technologies Inc., Enschede, The Netherlands) was fixed at the distal end of the cane using a jig fabricated using a 3D printer (Figure 1). Accelerations and angular velocities were sampled at 120 Hz. Before the measurement, the length of the cane was adjusted according to each participant’s height. The participants identified the side of the cane that was comfortable for them to hold. If they experienced pain or disability, they held the cane on the opposite side. They were allowed to sufficiently practice walking to become accustomed to their environment. Furthermore, they were instructed to walk 1–2 round trips on a 7 m straight path at a comfortable speed with a two-movement gait in which the cane and the opposite foot were simultaneously grounded. The number of measurements depended on the participant’s availability and the nature of the study conditions. For instance, if a participant was available for two round trips, we took measurements across these trials. Conversely, if a participant could attend only one round trip, we took a single measurement. Two trials were extracted from the round trip.

### 2.3. Data Analysis

**Figure 2:**
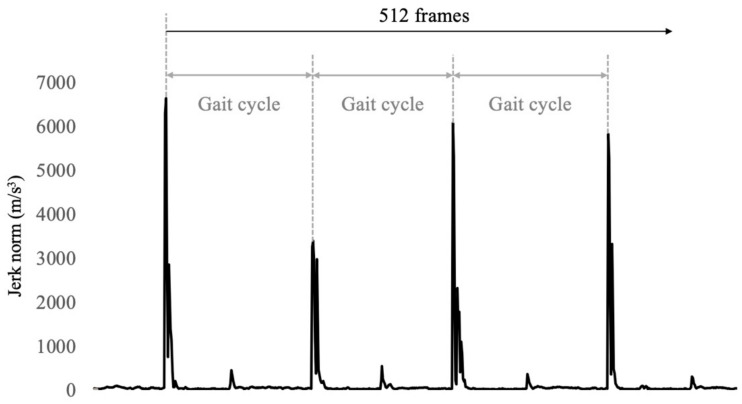
A typical example of time series data of jerk norm into measurements. The peak values indicate the ground contact of the cane. Peak value intervals are shown in walking cycles.

The IMU data were filtered using a fourth-order Butterworth low-pass filter with a cutoff frequency of 10 Hz [15]. The ground contacts were detected using the jerk norm according to a previous study [8]. The waveform of the jerk norm shows that a large spike occurs when the cane touches the ground (Figure 2). Subsequently, 512 frames were extracted from the time of the first cane ground contact. The gait cycle was defined as the period between the cane contact. Subsequently, we counted the gait cycle time. In each gait cycle, the root mean square (RMS) of the acceleration was calculated in the vertical (VT), anteroposterior (AP), and mediolateral (ML) directions. In addition, the peak angular velocity per gait cycle was extracted for each direction. The calculated values were averaged.

To calculate the power spectrum, we applied a 512-point Fast Fourier Transform to the filtered acceleration through the periodic Hamming windows. The mean power frequency (MPF) was calculated within the frequency band of 0.5–10 Hz.

### 2.4. Frailty Assessment

We used the KCL to assess physical frailty and extracted its exercise-related section [16], considering that gait patterns were the most associated with physical frailty. This section was composed of five items: “Do you normally climb stairs without using handrail or wall for support?”, “Do you normally stand up from a chair without any aids?”, “Do you normally walk continuously for 15 min?”, “Have you experienced a fall in the past year?”, and “Do you have a fear of falling while walking?”. Those who answered three or more negative answers to these five items were judged to have physical frailty.

### 2.5. Statistical Analysis

Before the dependent variables were compared, the normality assumptions of each data point were confirmed using the Shapiro–Wilk test [17]. Subsequently, the differences in the variables were compared between older adults with and without physical frailty, using the Mann–Whitney U test [18]. The effect size (r-value) was also calculated as the amplitude of the differences, which were classified as small (0.1 ≤ r < 0.3), moderate (0.3 ≤ r < 0.5), and large (0.5 ≤ r) [19]. Statistical significance was set at *p* < 0.05. All statistical data were analyzed using SPSS statistical software (version 29.0; SPSS Japan Inc., Chicago, IL, USA).

In this study, we created five machine learning models to classify physical frailty in older adults, using MATLAB R2023b (MathWorks, Inc., Natick, MA, USA), decision tree (DT), linear discriminant analysis (LDA), k-nearest neighbors (k-NNs), support vector machine (SVM) with Gaussian kernel, and random forest (RF) [20]. These models were developed on a Mac (macOS version 14.6) equipped with an Apple M1 Max processor, 64 GB of RAM, and 2 TB of storage.

- DT: A simple and highly interpretable model that allows for a visual understanding of feature importance. In this study, the number of splits was set to 5.
- LDA: Used to evaluate whether the data are linearly separable. LDA is computationally efficient and effective when strong linear relationships are present.
- k-NN: A non-parametric method that is well suited to capturing local patterns in the data. It directly leverages similarities between different physical frailty states. The datasets were normalized to z-scores for this model.
- SVM: Capable of handling non-linear data and effective in classifying high-dimensional data. Using a Gaussian kernel, it can effectively classify data with complex boundaries. The datasets were normalized to z-scores for this model as well.
- RF: An ensemble learning method that helps prevent overfitting and offers high classification performance. It also enables the evaluation of feature importance. In this study, the ensemble method was performed using the following parameters: LogitBoost as the method, 243 learning cycles, and a learning rate of 0.59256. This ensemble model combines multiple weak learners to improve classification performance.

Hyperparameters that minimized the tenfold cross-validation loss were determined through automatic hyperparameter optimization. The dataset was randomly divided into 10 equal subsets (folds). Each fold was used once as test data, while the remaining folds were used to train the model. Because the split was random, data from the same individual could have appeared in both the training and test sets. Each participant contributed data from multiple trials, and these trials were treated as independent instances in the analysis. The classification accuracy was calculated as 1 − misclassification rate, and area under the curve (AUC), precision, recall, and F1 score (weighted average of precision and recall) were averaged across the ten models obtained through cross-validation.

## 3. Results

Table 1 presents the characteristics of the participants. Out of the 48 older people enrolled, 3 were excluded because their data could not be measured. Ultimately, we included 37 trials from 14 older adults with physical frailty and 94 trials from 31 older adults without physical frailty. Demographic data showed no significant differences between the older adults with and without physical frailty.

**Figure 3:**
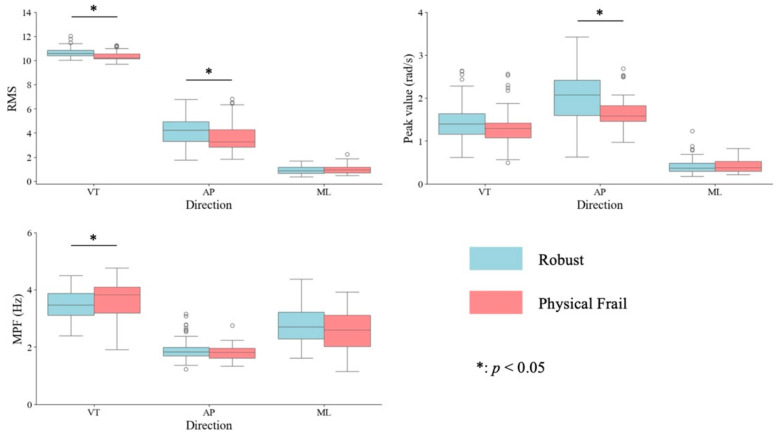
The box plots represent the root mean square (RMS) and mean power frequency (MPF) of acceleration and peak values of angular velocity in the vertical (VT), anteroposterior (AP), and mediolateral (ML) directions in older people with and without physical frailty. The box indicates the interquartile range. The horizontal line signifies the median, whereas the vertical lines represent the maximum and minimum values. The small circles indicate outliers. * indicates a significant difference between older people with and without physical frailty at *p* < 0.05.

Figure 3 and Table S1 show a comparison of the parameters obtained using the IMU built into the cane in older adults with and without physical frailty. The RMS in the VT and AP directions and the angular velocity in the AP direction were significantly smaller in older adults with physical frailty than in those without physical frailty (*p* < 0.001, r = 0.36; *p* < 0.001, r = 0.29; *p* < 0.001, r = 0.30, respectively). The MPF in the VT direction was significantly greater in older adults with physical frailty than in those without physical frailty (*p* = 0.042, r = 0.18). Moreover, the RMS in the ML direction and MPF in the AP and ML directions, the angular velocity in the VT and ML directions, and stride time were not significantly different between older adults with and without physical frailty (*p* = 0.50, r = 0.06; *p* = 0.543, r = 0.05; *p* = 0.111, r = 0.14; *p* = 0.177, r = 0.12; *p* = 0.971, r = 0.00; *p* = 0.427, r = 0.07).

**Figure 4:**
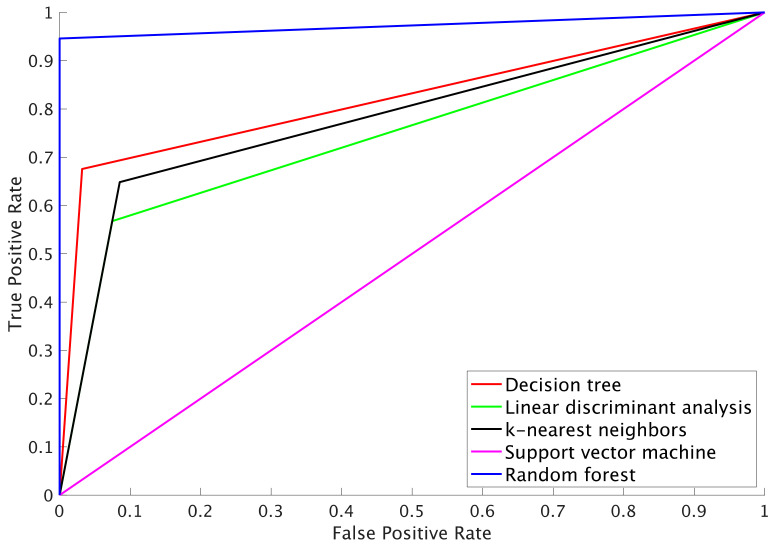
Receiver operating characteristic (ROC) curve of the five machine learning algorithms.

Table 2 summarizes the performance, Table 3 indicates the confusion matrix, and Figure 4 illustrates the receiver operating characteristic (ROC) curve of models in classifying physical frailty among older people. The classification accuracies of the models ranged from 71.8 for the SVM to 78.6 for the DT model. The F1 score ranged from 83.6 for the SVM and RF to 91.4 for the DT. The AUC ranged from 0.50 for SVM to 0.96 for RF. Among the five machine learning algorithms, the DT model was the most suitable for classifying physical frailty.

## 4. Discussion

This study examined the difference in cane use between older adults with and without physical frailty and created a classification model for physical frailty using the IMU built into the cane. The results showed that the characteristics of acceleration and angular velocity were significantly different and that the DT algorithm can classify older adults with and without physical frailty. Therefore, a cane with a built-in IMU can be utilized for the daily assessment of physical frailty in older people.

Older adults with physical frailty had significantly lower RMS and angular velocities in the AP direction than those without physical frailty. The RMS had approximately moderate effect sizes. Generally, stride length is shorter among older adults with physical frailty than among healthy older adults [21]. Hence, older adults with physical frailty have a decreased propulsive force during walking. Additionally, when the cane is grounded because of a small stride length, the posture angle is smaller [11]. Therefore, the forward momentum and swing of the cane decreased in older adults with physical frailty. These results were consistent with those of a previous study. Thus, in older adults with physical frailty, the ability to walk can be evaluated using the acceleration and angular velocity of the cane in the AP direction.

The small RMS of the cane in the VT direction reflects a small collision of the cane with the ground. At the initial contact, vertical ground reaction force is greater in a faster walking speed [22]. Therefore, collision intensifies when using a cane [11]. However, stride length is expected to be shorter in older adults with physical frailty than in those without physical frailty [23]. Thus, in our study, the RMS value in the VT direction became smaller among older adults with physical frailty than among the healthy ones.

In the frequency analysis, only the MPF in the VT direction of older adults with physical frailty was significantly smaller than that of older adults without physical frailty. According to Espy et al., compensating for the unstable walking patterns of older adults with physical frailty by reducing their stride length, which results in a shorter stride, seems logical [24]. For them, the cane becomes a stronghold to support their balance. In posture adjustment, the high-frequency component of time-series data reflects a reactive and adaptive balance strategy [25]. In this study, the MPF in the VT direction may have increased among older adults with physical frailty to support their unstable balance with a cane.

Among the five machine-learning models developed, the DT model demonstrated the highest efficacy in classifying physical frailty. This model could classify older people with physical frailty, with an accuracy rate of 78.6%, a precision of 96.8%, a recall of 88.3%, an F1 score of 92.4%, and an AUC of 0.82. Hence, the DT model had a very low rate of false positives, suggesting that it can reliably detect and correctly identify instances of physical frailty. Our metrics are comparable to the results of previous studies that classified physical frailty using an insole sensor [26,27] and sit-to-stand motion patterns [28]. In addition, a model with an AUC of 0.801–0.841 could be judged excellent [29]. The DT model can visually represent hierarchical interconnections among the parameters [20]. Considering the benefits of using an IMU-fitted cane to assess gait, the physical frailty of people using this cane can be evaluated daily.

On the other hand, although the RF also demonstrated large metrics, these results were obtained from the model using tenfold partitions, and its averaged accuracy was lower than that of the DT model. This indicates that the RF model may have a lower generalization performance and could overfit the data. Therefore, this study concluded that the DT model was the most suitable for classifying physical frailty from both performance and interpretability perspectives. Improving the performance of the RF model may require increasing the number of subjects, which should be addressed in future research.

This study had some limitations. First, this study did not collect objective biomechanical data, such as gait speed, kinematics, or the kinetics of walking. Thus, the differences in the RMS, angular velocity, and frequency characteristics measured with the cane were attributable to the differences in the gait of older adults with physical frailty. This limitation may have introduced a potential source of bias as more detailed biomechanical assessments could offer deeper insights into the underlying mechanisms of gait in this population. Future studies should aim to include detailed kinematic and kinetic measures, as well as comfortable walking speed and other spatiotemporal measures, to better understand the underlying mechanisms of gait differences between frail and non-frail older adults. Second, this study had a small sample size; thus, the results could not be generalized. Nevertheless, the differences in some parameters obtained through the IMU built into the cane had moderate effect sizes. Thus, if gait can be clearly assessed using the IMU built into the cane, developing this into a large cohort or longitudinal data measurement may be possible. Third, the participants in this study comprised both habitual cane users and non-users. Future studies should isolate the effects of physical frailty on gait characteristics without confounding factors of varying levels of experience with cane use. Fourth, only the exercise-related section of the KCL was used to determine the presence of physical frailty. This method does not provide a complete picture of physical frailty, which is a multi-dimensional condition [5]. Finally, this study used only the IMU to minimize the computational cost and weight impact. Previous studies have used a strain gauze and pressure sensor [8,9,30]. We were able to consider the type of sensor attached to the cane to improve the evaluation accuracy.

## 5. Conclusions

The characteristics of using a cane in older people with physical frailty include acceleration, angular velocity, and the acceleration’s frequency response in the VT and AP directions. These characteristics were used in creating a machine learning model to determine physical frailty. We ultimately found that the DT model demonstrated the most suitable performance based on evaluation metrics such as accuracy, precision, recall, and AUC. The DT model’s simplicity and interpretability make it highly practical for clinical settings, where understanding the decision-making process is crucial. This study suggests that physical frailty can be effectively assessed daily using an IMU attached to the cane in older adults.

However, this study has limitations, including limited sample size and data collected from a single facility, which may affect the generalizability of the results. Future work should involve validating the model with larger and more diverse datasets and exploring other machine learning methods and evaluation metrics to enhance model performance and clinical applicability.

## 6. Patents

The results of this study validate the recently published patent, “Frail state evaluation assist device, frail state evaluation assist system, frail state evaluation assist method, and frail state evaluation assist program”. JP Patent No. 7455263 was filed on 10 August 2023 and issued on 25 March 2024.

## Figures and Tables

**Table 1:** Demographic characteristics of participants.

	Total	Physical Frail	Robust	*p*-Value
Age (years)	81.0 (7.5)	85.0 (9.0)	80.0 (7.5)	0.063
Sex, *n* (%)				
Male	13 (28.2)	4 (26.6)	9 (30.0)	
Female	32 (71.8)	11 (73.4)	21 (70.0)	
Height (m)	1.53 (0.17)	1.52(0.07)	1.53 (0.12)	0.238
Weight (kg)	50.5 (11.6)	48.4 (20.6)	51.0 (10.6)	0.887
BMI (kg/m^2^)	22.3 (3.8)	22.4 (3.7)	22.5 (3.4)	0.198

Values: Median (interquartile).

**Table 2:** The model performance of the five machine learning algorithms.

	Decision Tree	Linear Discriminant Analysis	k-Nearest Neighbors	Support Vector Machine	Random Forest
Accuracy (%)	78.6	73.3	73.3	71.8	76.3
Precision (%)	96.7	92.1	90.6	100	99
Recall (%)	87.5	82.8	88.9	71.8	97.8
F1 score	91.8	87.0	89.7	83.6	98.3
AUC ^1^	0.81	0.72	0.81	0.5	0.96

^1^ AUC: area under curve.

**Table 3:** Confusion matrix of the five machine learning algorithms.

		Decision Tree		Linear Discriminant Analysis		k-Nearest Neighbors		Support Vector Machine		Random Forest	
		Actual		Actual		Actual		Actual		Actual	
		True	Negative	True	Negative	True	Negative	True	Negative	True	Negative
Predicted	True	90.9	13.0	86.6	18.0	85.2	10.6	94.0	37.0	93.1	2.2
	Negative	3.1	24.0	7.4	19.0	8.8	26.4	0.0	0.0	0.9	34.8

## Data Availability

Data available on request due to restrictions (ethical reasons).

## Supplementary Materials

The following supporting information can be downloaded at: https://www.mdpi.com/article/10.3390/s24216910/s1, Table S1: Values obtained by inertial measurement unit attached to a cane in older people with and without physical frailty.
