# Supplementary material for: Physical Frailty Prediction Using Cane Usage Characteristics during Walking
Source: Sensors (Basel). 2024 Oct 28;24(21):6910. doi: 10.3390/s24216910 (PMC11548610; doi:10.3390/s24216910)
Supplement: Supplementary file 1 [file sensors-24-06910-s001.zip › sensors-3220874-supplementary.pdf]

Supplemental Table S1. Values obtained by inertial measurement unit attached to a cane in older people with and without physical frailty.

		**Physical Frail**	**Robust**
RMS	VT	10.20 (10.16 – 10.54)	10.59 (10.42 – 10.85)
	AP	3.29 (2.77 – 4.27)	4.27 (3.40 – 4.94)
	ML	0.96 (0.67 – 1.18)	0.88 (0.66 – 1.16)
MPF	VT	3.82 (3.15 – 4.11)	3.47 (3.10 – 3.88)
	AP	1.81 (1.62 – 1.97)	1.81 (1.62 – 1.97)
	ML	2.59 (2.00 – 3.17)	2.71 (2.26 – 3.22)
Angular Velocity	VT	1.29 (1.06 – 1.59)	1.41 (1.15 – 1.65)
	AP	1.58 (1.45 – 1.87)	2.09 (1.59 – 2.44)
	ML	0.38 (0.28 – 0.53)	0.36 (0.30 – 0.48)

Values: Median (interquartile). RMS: Root mean square. MPF： Mean power frequency. VT: Vertical. AP: Antero-posterior. ML: Medio-lateral.
